# Motivational Interviewing as an Intervention to Improve Antiretroviral Treatment Initiation Among People who Inject Drugs (PWID): A Pilot Study in Jakarta and Bandung, Indonesia

**DOI:** 10.2174/2589977515666230531154629

**Published:** 2023-06-08

**Authors:** Evi Sukmaningrum, Astri Parawita Ayu, Lydia Verina Wongso, Miasari Handayani, Sarahsita Hendrianti, Nurhayati Hamim Kawi, Nur Aini Kusmayanti, Nurjannah Sulaiman, Irwanto, Matthew Law, Rudi Wisaksana

**Affiliations:** 1 University Centre of Excellence-AIDS Research Centre Health Policy and Social Innovation, Atma Jaya Catholic University of Indonesia, Jakarta, Indonesia;; 2 Faculty of Psychology, Atma Jaya Catholic University of Indonesia, Jakarta, Indonesia;; 3 School of Medicine and Health Sciences, Atma Jaya Catholic University of Indonesia, Jakarta, Indonesia;; 4 Research Center for Care and Control of Infectious Diseases, Faculty of Medicine, Padjadjaran University, Bandung, Indonesia;; 5 Faculty of Public Health, University of Indonesia, Depok, Indonesia;; 6 Center for Tropical Medicine, Faculty of Medicine, Public Health and Nursing, Universitas Gadjah Mada, Yogyakarta, Indonesia;; 7 Subdirectorate HIV&AIDS, Ministry of Health of the Republic of Indonesia, Surabaya, East Java, Indonesia;; 8 Kirby Institute, University of New South Wales, Sydney, Australia

**Keywords:** Motivational interviewing, HIV, injecting drug use, time-to-treatment, treatment adherence, healthcare, PWID, MI counseling, ART initiation

## Abstract

**Introduction:**

Progress towards the 95-95-95 target among People Who Inject Drugs (PWID) with Human Immunodeficiency Virus (HIV) infection was considerably low. A behavioral approach, such as motivational interviewing (MI), has been recognized as an effective strategy for improving HIV treatment outcomes among PWID.

**Objective:**

This study aimed at assessing the impact of MI counselling to improve ARV initiation among HIV-positive PWID.

**Methods:**

A cohort design pilot study was performed, and participants were recruited using a convenience sampling technique. Participants were PWID with HIV who accessed healthcare facilities in two Indonesian cities. Selected participants were assigned to an intervention group and a control group. The intervention group followed MI counselling, while the control group received ART following the standard of care. The participants were assigned to each group based on their preferences. The data was collected between January 2018 and January 2019.

**Results:**

In total, 115 PWID with HIV participated in this study in the intervention (n = 30) and control (n = 85) groups. All but one intervention group's participants started ART, while 68/85 in the control group did so. Receiving MI counselling significantly contributed to ART initiation. In addition, the participants were followed-up until 12 months after ARV initiation. During this period, we found that similar proportions of participants in both groups discontinued the treatment, and only a small number achieved HIV viral suppression.

**Conclusion:**

The positive effect of MI counselling on ART initiation provides insight into the possibility of its wider implementation. Further studies are needed to gain a deeper understanding of MI counselling and its effect on other outcomes of the HIV treatment cascade.

## 1 INTRODUCTION

The Global UNAIDS data have shown that as of 2021, People Who Inject Drugs (PWID) have 35 times the risk of new infections [1]. Progress towards the 95-95-95 target the UNAIDS sets is considerably low among PWID with HIV [1, 2]. This target requires 95% of people living with HIV (PLWH) to know their HIV-positive status, 95% of those diagnosed with HIV to receive and be retained in treatment, and 95% of PLWH who receive treatment to achieve viral suppression. Indonesia is still far from achieving this target. The Indonesian Ministry of Health report showed that at the end of 2020, 60% of PLWH knew their HIV-positive status, about 47% of them had initiated care, about 26% were on ART, and only 6.1% were virally suppressed [3]. In Indonesia, the proportion of PWID who initiated and retained in ART was even lower than that of other key populations (men who have sex with men, female sex workers, and transgender people) [4]. This data was shown by the HIV Early Testing and Treatment in Indonesia (HATI) study, in which 51 PWID were recruited as participants. They found that only 67% had started ART, and 55% remained on treatment after six months [4]. A systematic review showed that among the included studies from India, US, and Canada, the percentages of PWID on ART ranged between 20%-73%, whereas the proportions of those who retained in care were 25%-84% [5]. All the above-mentioned data show an urgent need to improve ART initiation among PWID. Behavioral interventions, such as counselling and peer support, have been suggested as an approach to improve the linkage to HIV care in low and middle-income countries [6]. The HPTN 074 study conducted in Ukraine, Vietnam, and Indonesia has shown that an integrated intervention can improve treatment outcomes, including ART adherence and achieving viral suppression [7].

One behavioral intervention that has been applied to improve ART adherence is motivational interviewing (MI) [8]. It is a collaborative and conversational counselling approach that strengthens clients’ motivation and commitment to change their behavior [9]. MI was developed based on the theory of change; it adopts a collaborative conversation style and focuses on the client’s internal values and goals in encouraging the clients’ motivation and commitment to change their behaviors [9]. The client is assisted in the process of change, which includes several stages based on the theory of change: pre-contemplation, contemplation, preparation, action, and maintenance. MI counselling has proven effective in reducing risky behaviors, including substance use, and improving mental health and treatment adherence among patients, including PLWH [10-12].

Studies showed positive effects of MI counselling, significantly better than various types of health education programs and the standard of care, on HIV treatment adherence among PLWH from various backgrounds [11]. For example, a significant improvement in ART adherence was found in studies involving PLWH, including women [13, 14] and injecting drug users [15]. This positive effect also was found when the MI counselling was delivered in a group [16] or combination with other psychosocial counselling approaches [7]. Since MI counselling has shown positive effects on HIV treatment adherence, it can be assumed that it may also be beneficial for ARV initiation. However, such an assumption needs to be further proven.

We undertook a prospective cohort study in Bandung and Jakarta, Indonesia, to assess the impact of counselling using the MI approach to improve ART initiation among HIV-positive PWID. In addition, the retention rate and viral load (VL) suppression were measured to explore the potential further effects of MI counselling on outcomes of the HIV care cascade. To the best of our knowledge, this is the first study in Indonesia that evaluates the implementation of MI counselling for such a purpose. We hypothesized that MI counselling, compared to standard of care, would improve the likelihood of ART initiation among PWID with HIV.

## 2 MATERIALS AND METHODS

### 2.1 The HATI Study

The HATI study intervention phase (ClinicalTrials.gov identifier: NCT03659253) was performed to explore and apply strategies in improving the outcomes of the HIV care cascade among key populations of PLWH in four cities of Indonesia, namely Bandung (West Java Province), Jakarta (DKI Province), Yogyakarta (DIY Province), and Denpasar (Bali Province). The observational cohort phase of the HATI study was performed previously and has been reported elsewhere [4]. The current study was part of the intervention phase of the HATI project, in which MI counseling was implemented in Jakarta and Bandung. During the observational phase in these two cities, PWID participants were recruited from 15 health facilities. Four of them were selected afterward as the sites for implementing the MI intervention. These sites were one outpatient HIV clinic of a general hospital and one primary health care (Bandung), and two primary health care units (Jakarta). However, one site in Bandung then dropped out as no PWIDs with HIV accessed it.

### 2.2 Study Design and Participants

**Fig. (1):**
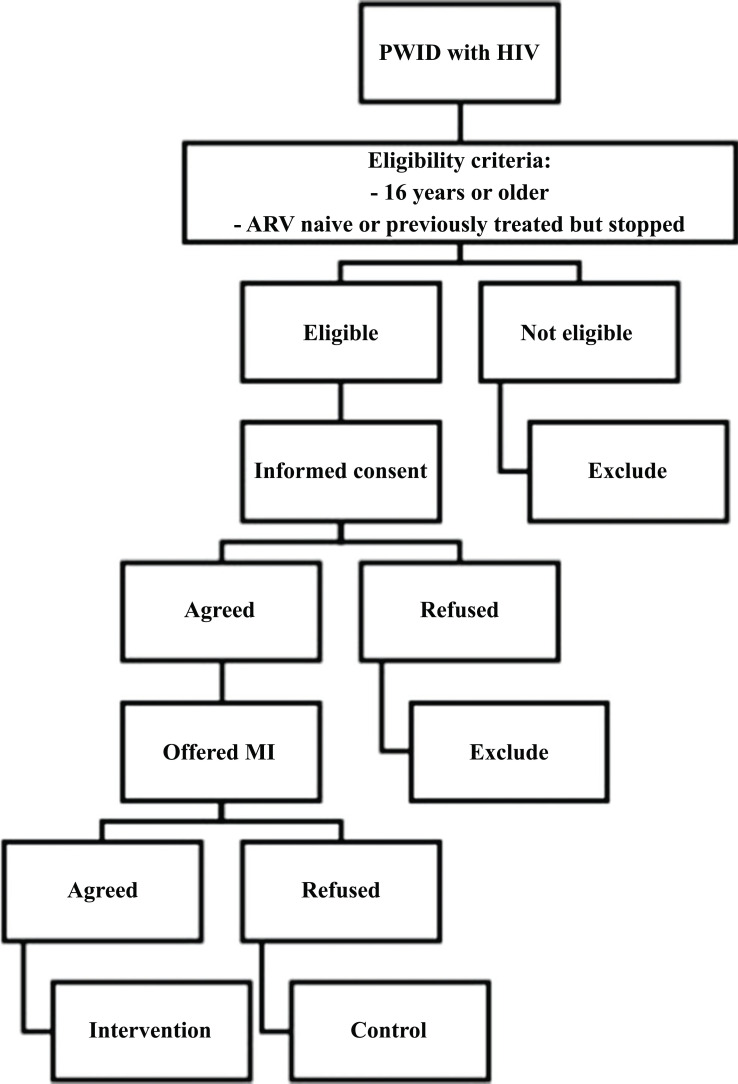
Flow diagram of the recruitment process.

This pilot study used a cohort design and a convenience sampling technique to recruit the participants. The eligibility criteria for inclusion were PWID with HIV aged 16 years or older who accessed the HIV clinic of the above-mentioned facilities and had never started ART (naïve) or had initiated it but then discontinued (previously treated). The participants were recruited from 14 study sites in Bandung and Jakarta. Among them, four were chosen as the intervention sites because they had the highest number of PWID with HIV patients during the observational phase of the study. No cash compensation was given for participating in this study, but free VL tests were provided during the study period. In Indonesia, a free VL test program was unavailable during this study. Eligible patients in the intervention sites were given information about the HATI study and were offered to participate by receiving MI counselling. Those who agreed to participate had the MI procedure explained. Participants were required to provide written consent to enroll in the study and avail counselling. They were to attend a minimum of four and a maximum of 10 counselling sessions over 12 months, and all sessions were recorded. The data was collected between January 2018 and January 2019. The control group consisted of the HATI study participants recruited from the non-intervention sites in Jakarta and Bandung during the observational cohort phase [4]. The participants in the intervention sites who declined MI were also included in the control group. All participants were asked to sign a written informed consent beforehand. The recruitment process is depicted in the flow diagram (Fig. **1**). The Primary Investigator (ES) and other research team members (LVW, MH, RW) recruited the participants. The report of this study was written following the Strengthening the Reporting of Observational Studies in Epidemiology (STROBE) [17, 18]. The STROBE checklist is provided in Table **S1**.

### 2.3 Outcomes

The primary outcome was initiating ART within 1 year after starting MI counselling (intervention) or being recruited (control). The healthcare workers reported the participants who started ART; the number was documented. Although the main purpose of the counselling was to enhance ART initiation, the counselling was continued until 12 months, regardless of ART initiation. Through this method, we aimed to gain insight into the potential effects of counselling in keeping participants on ART and, subsequently, achieving viral suppression. The participants who initiated ART were followed up monthly for the next 12 months. The number of participants who stayed on and left ART during this period was documented. Those who left treatment were contacted and encouraged to return. The HIV viral load (VL) level after ART initiation was measured to determine viral suppression (VL≤1000 copies/ml) [19]. The measurement was performed within the period of 6 to 12 months after starting ART. Since MI counselling aimed primarily at improving the motivation to initiate ART, viral suppression was evaluated as an additional outcome. Other factors that might influence the MI effect were identified. These factors were age, education level, employment status, marital status, HIV stage, and ART status.

### 2.4 Data Analysis

Demographic characteristics were analyzed descriptively to calculate the proportion of participants based on age group, education level, employment status, and marital status. The proportion of participants with low (stages 1 and 2) or high (stages 3 and 4) HIV stage and in each ART status (naïve or previously treated) at recruitment was also calculated. The Kaplan-Meier survival and Cox regression analyses were performed to evaluate the main outcome (the effect of MI counselling on ART initiation). Kaplan-Meier survival was used to compare ART initiation between groups visually, and Cox regression was used for formal comparisons. Cox regression was also performed to determine predictors of ART initiation (the participation in the MI counselling, HIV stage and ART status at recruitment, and demographic characteristics).

As additional analyses, the same methods were used to compare the failure to remain in treatment between groups and evaluate its predictors. Whereas logistic regression analysis was performed to determine variables contributing to VL suppressions. The predictors were participation in the MI counselling, HIV stage and ART status at recruitment.

## 3 EXPERIMENTAL

MI counselling was the intervention implemented in the current study. The main purpose of the counselling was to enhance the participants’ motivation to initiate ART. In addition, the potential effect of MI counselling on the continuation of the treatment and VL suppression was explored. In this study, MI counselling was to be delivered in a minimum of four and a maximum of ten counselling sessions over 12 months. Counselling was carried out by healthcare workers in the intervention sites. Several strategies were established to aid them in providing counselling. First, they were required to follow a series of MI training sessions prior to the commencement of the study. Second, a module was developed and tried out as guidance on providing MI counselling in an outpatient health care setting. Finally, the healthcare workers were supported by a trained psychologist. The psychologist accompanied them in the first few sessions and assisted them in dealing with any issue concerning the counselling delivery. The MI module comprises two parts. The first part describes topics for those who are in the early stages of change (pre-contemplation, contemplation, and preparation), while the second one describes topics for those in later stages (action and maintenance). In this study, the decisions on which part and topic a participant would start were determined based on their ART status. All participants who were ART naïve and previously lost to follow-up after ART initiation commenced the MI counselling from the first part of the module. The participants on ART could start with either the first or second module based on their stages of change in ART adherence. More detailed information about the MI modules, the healthcare workers and the MI training are given in the Table **S2**.

## 4 RESULTS

### 4.1 Characteristic of Participants

In total, 30 and 85 participants were recruited from the intervention and the control sites, respectively (Table **1**). Only six females participated in the intervention (6.7%) and the control (4.7%) groups. The age of participants ranged from 22 to 61 years old (mean of age = 35, SD = 5.6). Most participants were high school and higher education graduates (90.0% in the intervention group and 69.4% in the control group), currently married (40.0% in the intervention group and 42.4% in the control group). They had jobs (76.7% in the intervention group and 64.7% in the control group). There was a significant difference in education level (*p* = 0.03) and baseline ART status (*p* <0.001) between participants in the intervention and control groups. The demographic characteristics of the participants are described in the Table **2**.

### 4.2 ART Initiation

**Fig. (2):**
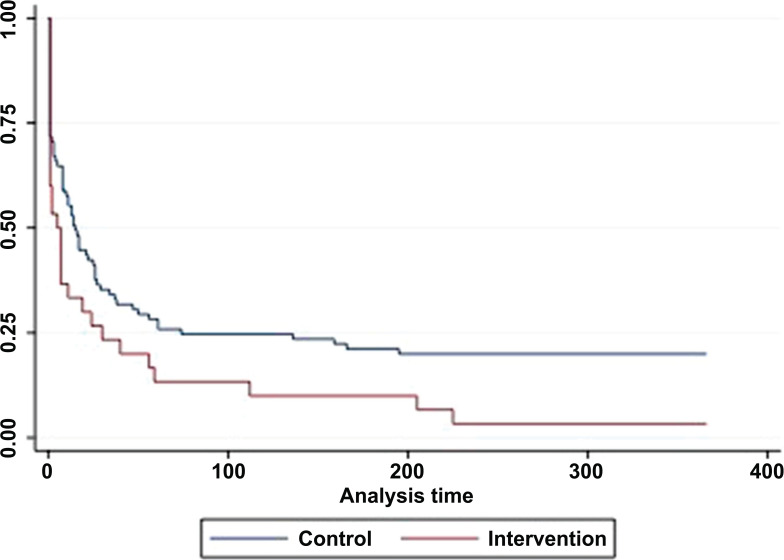
Time to ART initiation.

The ART initiation rate was 96.7% (29 of 30) in the intervention group and 80.0% (68 of 85) in the control group (*p* = 0.03). The Kaplan-Meier curves show that 50% of the intervention group participants started ART on day 6 after they were recruited, whereas 50% of the control group participants started ART on day 15 after they were recruited (Fig. **2**).

The univariate analysis revealed that the participants in the intervention group were significantly more likely and sooner to start the ART than the control group (HR = 1.58; 95% CI = 1.02-2.44; *p* = 0.04) (Table **3**). However, when including other variables in multivariate analysis, the significance decreased (*p* = 0.08). Another variable close to statistically significant was marital status, which revealed that those who were divorced were less likely to start the ART than those who were single or never married (HR = 0.5; 95% CI = 0.3-1.0; *p* = 0.04). There were no other variables that significantly influenced ART initiation.

### 4.3 Treatment Failure

**Fig. (3):**
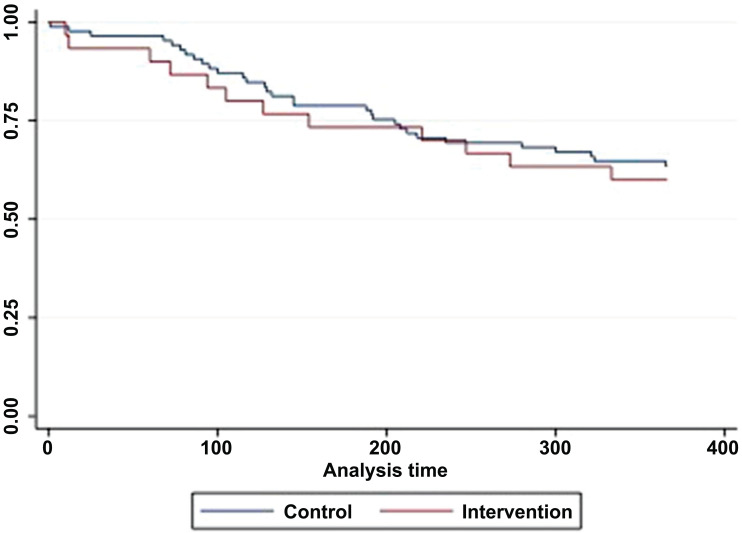
Time to treatment failure.

Within 12 months after starting the treatment, only 55.7% (17 out of 29 in the intervention group and 37 out of 68 in the control group) of those starting ART remained in treatment. The participants who did not remain across both groups were 15.5% deaths, 2.1% referred to other health facilities, and 26.8% did not come to collect the medicine two months in a row. The Kaplan-Meier analysis revealed 50% of participants in each group left treatment in 1 year after being recruited (Fig. **3**).

All participants who discontinued were included in the analysis. HIV stages at recruitment were the only significant predictor for failure to remain in the treatment (Table **S3**). The participants with HIV stage 3 or 4 at recruitment were more likely to leave treatment than stage 1 or 2 (HR=2.4, 95% CI=1.2-4.8, *p* = 0.01). No other variables significantly predicted the failure. Among the intervention group participants who started the ART, 75.86% (22 out of 29) received the MI sessions according to the protocol (four sessions or more). The other 24.14% (7 out of 29) received only 3 sessions, and subsequently, all failed to remain in treatment at 12 months. In contrast, only 22.73% (5 out of 22) of those who followed four or more sessions did not remain.

### 4.4 Viral Suppression

Not all participants who started the ART appeared for a VL test within the 6- to 12-month window. Among 97 participants who initiated ART, six left treatment before the period of the VL test. Subsequently, 36.26% (33 out of 91) of them did a VL test. As such, only this number was included in calculating the viral suppression rate. In total, 81.82% of those who tested for VL achieved viral suppression (intervention: 9 out of 12; control: 18 out of 21). The intervention group was less likely, although not significant, to be virally suppressed than the control group. HIV stage at recruitment did not have any effect on viral suppression. When recruited to this study, ART status was close to being statistically significant, associated with viral suppression, with those previously treated were less likely to achieve viral suppression (*p* = .07) (Table **S4**).

## 5 DISCUSSION

This is the first pilot study to evaluate the implementation of MI counselling in healthcare facilities in Bandung and Jakarta, Indonesia. Specifically, we aimed to assess the effect of MI counselling on ART initiation among PWID with HIV. Our analysis further showed that receiving MI counselling significantly increased ART initiation among PWID. All but one participant who followed counselling initiated ART, while 68/85 in the control group did so. In addition, the participants were followed up until 12 months of ART. During this period, we found that similar proportions of participants in both groups who started ART discontinued the treatment, and only a small number received viral load tests and were documented as achieving viral suppression. Low motivation has been suggested as one of the barriers for PWID to start ART [20]. Therefore, our finding addresses this issue since MI counselling aims to improve the patient’s motivation for HIV treatment. The MI counselling topics focus on helping participants to assess and to understand their motivation concerning the treatment. Furthermore, lack of information about HIV and the benefits of treatment were also identified as barriers to ART initiation [20, 21]. Information about living with HIV infection and the effects of ART on an individual’s personal life was provided in the MI counselling. Having such information can, we believe, empower PWIDs to be more responsible for their own lives and to set a goal concerning their health. This might help them to increase their motivation to start ART. Another barrier to ART initiation recognized by service providers and PWID is stigma from healthcare providers [20, 22]. In MI counselling, a client is positioned as the center of treatment, and a counsellor must be empathetic and accepting. This method would diminish stigma and encourage PWID to start ART immediately. Indeed, it has been suggested that MI spirits, such as collaboration, encouraging rather than installing motivation and honoring the client’s autonomy, lead to a more positive outcome for the client [8-10]. MI counselling has been mostly used to enhance patients’ adherence to ART [8, 10, 23]. Studies that evaluated the effect of MI intervention on treatment adherence usually involved participants who were already on ART and had no information about ART initiation. Therefore, our study provides new insight concerning the benefit of MI counselling. Applying the MI approach to increase ART intake has also been considered by Mokhele *et al*. [24]. They described developing a readiness process model to guide the counsellor to apply MI counselling for ART initiation and retention in South Africa. Considering that many countries are still struggling with ART coverage, performing more studies to assess the effect of MI counselling on ART uptake will benefit them. Our analysis also showed similar proportions of participants in the intervention and control groups who discontinued the treatment within 12 months after initiating the ART. Furthermore, only a small fraction of participants in both groups had a VL test and were subsequently confirmed as virally suppressed. These results imply that MI counselling did not sufficiently help the participants to remain in the treatment or achieve viral suppression. This finding is related to the nature of PWID, and most of them still have issues with their addiction that might be a barrier to continuing the treatment. Considering the main purpose of the counselling was to improve the ART initiation, this finding was not completely unpredicted, although it should have been anticipated. However, it is also important to emphasize that failure in the intervention group seems to be correlated with the number of counselling sessions they followed. All participants who received only three sessions left treatment within 12 months and therefore did not have a VL test. Whereas only a small fraction of those who followed four or more sessions ceased ART, and more had a VL test. As such, these findings also suggest that the benefits of MI counselling may be enhanced by increasing the frequency of the sessions provided. Indeed, it has been suggested previously that a higher number of sessions is associated with better outcomes in behavioral changes, including ART adherence [10, 25]. Nevertheless, it is necessary to evaluate this further in future research.

## 6 LIMITATIONS

Our study also has some other limitations. Hence, the results may not be generalized or fully reflect other PWID conditions in Indonesia. Considering the limited number of PWID in Jakarta and Bandung, we needed to adjust the eligibility criteria by including the previously treated patients and those who were ART naïve. The limited number of PWID also made it impossible to do randomization. As such, we chose 4 sites with the largest number of PWID patients. Therefore, selection bias could not be ruled out. By the end of the recruitment period, we could only recruit a small number of PWID, especially in the intervention group. All of these conditions may influence the analysis and reduce power, although the ratio between the intervention and control group was still acceptable [26]. In addition, the intervention group participants agreed to receive the MI counselling because we needed their consent to conduct counselling. Therefore, there was also a selection bias that might influence the results. Some participants dropped out in both groups, which may influence the findings regarding the analysis that compares the proportion of viral suppression between groups. Future studies should consider better strategies to recruit more participants because of Indonesia's limited number of PWID [27]. Finally, we included all participants who discontinued treatment despite the reasons. As such, we also included those who died, which may influence the accuracy concerning the predictors of treatment failure. We also did not have information about the reason for death. Consequently, we could not do further analysis regarding this condition. We suggest that future studies consider the cause of death among participants in evaluating treatment failure to better understand this issue.

## CONCLUSION

In conclusion, despite its limitations, our study was the first that attempted to evaluate the effects of MI counselling for HIV-positive PWID in the primary healthcare setting in Indonesia. The positive effect of counselling on ART initiation provides insight into the possibility of its implementation. However, further studies are needed to gain a deeper understanding of the effects and cost-effectiveness of MI counselling on other outcomes of the HIV treatment cascade among key populations.

## Figures and Tables

**Table 1:** Study sites.

**S. No.**	**Sites**	**Intervention (n, %)**	**Control (n, %)**
1.	Hasan Sadikin Province Hospital	20 (61.8)	26 (27.1)
2.	Puskesmas Ibrahim Adjie	-	12 (12.5)
3.	Cengkareng District Hospital	-	9 (9.4)
4.	Puskesmas Senen	7 (29.4)	7 (7.3)
5.	Puskesmas Cengkareng	-	7 (7.3)
6.	Puskesmas Tambora	-	7 (7.3)
7.	Puskesmas Tanah Abang	-	6 (6.3)
8.	Puskesmas Taman Sari	-	4 (4.2)
9.	Puskesmas Gambir	-	4 (4.1)
10.	Bungsu Hospital	-	4 (4.1)
11.	Puskesmas Pasundan	-	4 (4.1)
12.	Puskesmas Grogol Petamburan	3 (8.8)	4 (4.1)
13.	Ujung Berung District Hospital	-	2 (2.1)
14.	Mawar Clinic	-	2 (2.1)
-	-	**30 (100)**	**85 (100)**

**Table 2:** Demographic characteristics, by group.

**-**	**-**	**Intervention (n, %)**	**Control (n, %)**	***p***
City	Bandung	20 (66.7)	39 (45.9)	0.05
-	Jakarta	10 (33.3)	46 (54.1)	
Gender	Male	28 (93.3)	81 (95.3)	0.68
-	Female	2 (6.7)	4 (4.7)	
Age Group	≤35 years	13 (43.3)	52 (61.2)	0.09
-	>35 years	17 (56.7)	33 (38.8)	
Education	Lower education (junior high school and below)	3 (10)	26 (30.6)	0.03*
-	Higher education (high school and above)	27 (90)	59 (69.4)	
Employment	Employed	3 (10)	26 (30.6)	0.23
-	Unemployed/student	27 (90)	59 (69.4)	
Marital Status	Single (Never married)	9 (30)	29 (34.1)	0.77
-	Currently married	12 (40)	36 (42.4)	
-	Divorced/widowed	9 (30)	20 (23.5)	
HIV Stage (baseline)	Stage 1-2	16 (53.3)	32 (37.7)	0.13
-	Stage 3-4	14 (46.7)	53 (62.4)	
ARV Status at baseline	Naïve	10 (33.3)	66 (77.7)	<0.001*
-	Previously treated	20 (66.7)	19 (22.4)	

**Note:** *Significant difference (*p* value <0.05).

**Table 3:** Factor associated with ART initiation.

**-**	**ART Initiation**		**Univariate Analysis**				**Multivariate Analysis**			
	**n**	**%**	**HR**	**95% CI**		***p***	**HR**	**95% CI**		***p***
**MI Counselling**										
Yes	29	96.7	1	-	-	-	1	-	-	-
No	68	80	1.6	1.0	2.4	0.04*	1.6	0.9	2.7	0.08
**HIV Stage at Recruitment**										
Stage 1 and 2	38	79.2	1	-	-	-	1	-	-	-
Stage 3 and 4	59	88.1	1.2	0.8	1.8	0.4	1.2	0.8	1.9	0.4
**ART Status at Recruitment**										
Naïve	61	80.3	1				1			
Previously treated	36	92.3	1.3	0.9	2.0	0.2	1.4	0.8	2.2	0.2
**Gender**										
Male	92	84.4	1	-	-	-	1	-	-	-
Female	5	83.3	0.9	0.4	2.3	0.9	0.8	0.3	2.1	0.7
**Age Group**										
Below 35	56	86.2	1	-	-	-	1	-	-	-
Above 35	41	82.0	1.0	0.7	1.6	0.8	1.1	0.7	1.8	0.6
**Education Group**										
Lower education (junior high school and above)	23	79.3	1	-	-	-	1	-	-	-
Higher education (high school and above)	74	86.1	1.1	0.7	1.8	0.7	1.1	0.6	1.8	0.8
**Employment**										
Unemployed	33	89.2	1	-	-	-	1	-	-	-
Employed	64	82.1	0.9	0.6	1.4	0.7	0.9	0.6	1.3	0.5
**Marital Status**										
Single (never married)	35	92.1	1	-	-	-	1	-	-	-
Currently married	40	83.3	0.9	0.6	1.5	0.8	1.0	0.6	1.5	0.8
Widowed/divorced	22	75.9	0.7	0.4	1.1	0.1	0.5	0.3	1.0	0.04*

**Note:** *Significant difference (*p* value <0.05).

## Data Availability

The data supporting this study's findings are available from the corresponding author, APA, upon reasonable request.

## LIST OF ABBREVIATIONS

HIV: Human Immunodeficiency Virus
MI: Motivational Interviewing
PWID: People Who Inject Drugs
STROBE: Strengthening the Reporting of Observational Studies in Epidemiology
VL: Viral Load

## STANDARDS OF REPORTING

STROBE guidelines were followed.
