# Supplementary material for: Motivational Interviewing as an Intervention to Improve Antiretroviral Treatment Initiation Among People who Inject Drugs (PWID): A Pilot Study in Jakarta and Bandung, Indonesia
Source: Curr Drug Res Rev. 2023 Jun 8;16(2):228–36. doi: 10.2174/2589977515666230531154629 (PMC11340277; doi:10.2174/2589977515666230531154629)
Supplement: Supplementary file 1 [file CDRR-16-228_SD1.pdf]

SUPPLYMENTARY MATERIALS

Motivational Interviewing as an Intervention to Improve Antiretroviral Treatment Initiation Among People who Inject Drugs (PWID): A Pilot Study in Jakarta and Bandung, Indonesia

Evi Sukmaningrum[1,2], Astri Parawita Ayu[1,3,*], Lydia Verina Wongso[1], Miasari Handayani[4], Sarahsita Hendrianti[1], Nurhayati Hamim Kawi[5], Nur Aini Kusmayanti[6], Nurjannah Sulaiman[7], Irwanto Irwanto[1], Matthew Law[8] and Rudi Wisaksana[4]

[1]University Centre of Excellence AIDS Research Centre Health Policy and Social Innovation, Atma Jaya Catholic University of Indonesia, Jakarta, Indonesia; [2]Faculty of Psychology, Atma Jaya Catholic University of Indonesia, Jakarta, Indonesia; [3]School of Medicine and Health Sciences, Atma Jaya Catholic University of Indonesia, Jakarta, Indonesia; [4]Research Center for Care and Control of Infectious Diseases, Faculty of Medicine, Padjadjaran University, Bandung, Indonesia; [5]Faculty of Public Health, University of Indonesia, Depok, Indonesia; [6]Center for Tropical Medicine, Faculty of Medicine, Public Health and Nursing, Universitas Gadjah Mada, Yogyakarta, Indonesia; [7]Subdirectorate HIV&AIDS, Ministry of Health Republic Indonesia; [8]Kirby Institute, University of New South Wales, Sydney, Australia

Supplementary Table 1. STROBE statement.

	Item No.	Recommendation	Page No.	Relevant text from manuscript
**Title and abstract**	1	(*a*) Indicate the study's design with a commonly used term in the title or the abstract	-	-
		(*b*) Provide in the abstract an informative and balanced summary of what was done and what was found	1	
		Introduction		
Background/rationale	2	Explain the scientific background and rationale for the investigation being reported	1-2	In Indonesia, the proportion of PWID who initiated and retained in ART was even lower than that of other key populations (men who have sex with men, female sex workers, transgenders).<br>Studies showed positive effects of MI counselling, which was better than various types of health education program and the standard of care, on HIV treatment adherence among PLWH from various background.

Objectives	3	State specific objectives, including any prespecified hypotheses	2	We undertook a prospective cohort study in Bandung and Jakarta Indonesia, to assess the impact of counselling using the MI approach to improve ART initiation among HIV-positive PWID.<br>We hypothesized that MI counselling, compared to standard of care, would improve the likelihood of PWID with HIV to initiate ART.
		Methods		
Study design	4	Present key elements of study design early in the paper	2	This was a pilot study using a cohort design and a convenience sampling technique to recruit the participants.
Setting	5	Describe the setting, locations, and relevant dates, including periods of recruitment, exposure, follow-up, and data collection	2	The participants were recruited from 14 study sites in Bandung and Jakarta. Among them, four were chosen as the intervention sites because they had the highest number of PWID with HIV patients during the observational phase of the study. Eligible patients in the intervention sites were given information about the HATI study and offered to participate by receiving MI counselling. They were to attend a minimum of four and a maximum of 10 counselling sessions over 12 months, and all sessions were recorded. The data was collected between January 2018 and January 2019.
Participants	6	(*a*) *Cohort study*—Give the eligibility criteria, and the sources and methods of selection of participants. Describe methods of follow-up.	2	The eligibility criteria for inclusion were PWID with HIV aged 16 years or older who accessed the HIV clinic of the above-mentioned facilities and had never started ART (naïve) or had initiated it but then stopped (previously treated). The participants were recruited from 14

				study sites in Bandung and Jakarta. Eligible patients in the intervention sites were given information about the HATI study and offered to participate by receiving MI counselling. Those who agreed to participate had MI procedure explained. Participants were required to provide written consent to enrol in the study and follow the counselling. The control group consisted of the HATI study participants recruited from the non-intervention sites in Jakarta and Bandung during the observational cohort phase.
		(*b*) *Cohort study*—For matched studies, give matching criteria and number of exposed and unexposed	2-3	The eligibility criteria for inclusion were PWID with HIV aged 16 years or older who accessed the HIV clinic of the above-mentioned facilities and had never started ART (naïve) or had initiated it but then stopped (previously treated). Those who agreed to participate had MI procedure explained. The control group consisted of the HATI study participants recruited from the non-intervention sites in Jakarta and Bandung during the observational cohort phase.<br><br>In total 30 and 85 participants recruited from the intervention and the control sites, respectively.
Variables	7	Clearly define all outcomes, exposures, predictors, potential confounders, and effect modifiers. Give diagnostic criteria, if applicable	3	The primary outcome was initiation of ART within 1 year after starting MI counselling (intervention) or being recruited (control).<br><br>The participants who initiated ART were followed up monthly for the next 12 months. The HIV

				viral load (VL) level after ART initiation was measured to determine viral suppression (VL≤1000 copies/ml). Other factors that might influence the MI effect were identified. These factors were age, education level, employment status, marital status, HIV stage, and ART status
Data sources/ measurement	8*	For each variable of interest, give sources of data and details of methods of assessment (measurement). Describe comparability of assessment methods if there is more than one group	3	The healthcare workers reported the participants who started ART and the number was documented. The number of participants who stayed on and left ART during this period was documented. The HIV viral load (VL) level after ART initiation was measured to determine viral suppression (VL≤1000 copies/ml).
Bias	9	Describe any efforts to address potential sources of bias	-	-
Study size	10	Explain how the study size was arrived at	-	-
Quantitative variables	11	Explain how quantitative variables were handled in the analyses. If applicable, describe which groupings were chosen and why	3	Demographic characteristics were analysed descriptively to calculate the proportion of participants based on age group, education level, employment status, and marital status. The proportion of participants with low (stage 1 and 2) or high (stage 3 and 4) HIV stage and in each ART status (naïve or previously treated) at recruitment was also calculated. Demographic characteristics, HIV stage, and ART status were evaluated to determine significant predictors of ART initiation and failure to remain in treatment. Additionally, the proportion of participants who left the treatment within 12 months and virally suppressed participants were calculated
Statistical methods	12	(*a*) Describe all statistical methods, including those used to control for confounding	3	Kaplan-Meier survival was used to compare ART initiation between groups

				visually, and cox regression was used for formal comparisons. Cox regression was also performed to determine predictors of ART initiation (the participation in the MI counselling, HIV stage and ART status at recruitment, and demographic characteristics). The same methods were also used for failure to remain in treatment and to evaluate its predictors. In addition, logistic regression analysis was performed to determine variables that contribute to VL suppressions. The predictors were participation in the MI counselling, and HIV stage and art status at recruitment.
		(*b*) Describe any methods used to examine subgroups and interactions	N/A	-
		(*c*) Explain how missing data were addressed	N/A	-
		(*d*) *Cohort study*—If applicable, explain how loss to follow-up was addressed	3	The number of participants who stayed on and left ART during this period was documented. Those who left treatment were contacted and encouraged to return.
		(*e*) Describe any sensitivity analyses	-	-
		Results		
Participants	13*	(a) Report numbers of individuals at each stage of study—eg numbers potentially eligible, examined for eligibility, confirmed eligible, included in the study, completing follow-up, and analysed	3	In total 30 and 85 participants recruited from the intervention and the control sites, respectively.
		(b) Give reasons for non-participation at each stage	-	-
		(c) Consider use of a flow diagram	3	
Descriptive data	14*	(a) Give characteristics of study participants (eg demographic, clinical, social) and information on exposures and potential confounders	3	Only six females participated in both the intervention (6.7%) and the control (4.7%) group. The age of participants ranged from 22 to 61 years old (mean of age=35, SD=5.6). Most participants were high school and higher education graduates (90.0% in the intervention group and 69.4% in the control group), currently married (40.0% in the intervention group and 42.4% in the control group) and had a

				job (76.7% in the intervention group and 64.7% in the control group).
		(b) Indicate number of participants with missing data for each variable of interest	N/A	
		(c) *Cohort study*—Summarise follow-up time (eg, average and total amount)	4	Within 12 months after starting the treatment, only 55.7% (17 out of 29 in the intervention group and 37 out of 68 in the control group) of those starting ART were retained in treatment.
Outcome data	15*	*Cohort study*—Report numbers of outcome events or summary measures over time	4	The Kaplan-Meier curves show that 50% of the intervention group participants has started ART on day 6 after they were recruited, whereas 50% of the control group participants had started ART on day 15 after they were recruited.<br>Within 12 months after starting the treatment, only 55.7% (17 out of 29 in the intervention group and 37 out of 68 in the control group) of those starting ART were retained in treatment.
Main results	16	(*a*) Give unadjusted estimates and, if applicable, confounder-adjusted estimates and their precision (eg, 95% confidence interval). Make clear which confounders were adjusted for and why they were included	4	The univariate analysis revealed that the participants in the intervention group were significantly more likely and sooner to start the ART compared to the control group (HR=1.58; 95%CI=1.02–2.44; p=0.04). However, when including other variables in multivariate analysis, the significance decreased (p=0.08). Another variable close to statistically significant was marital status, which revealed that those who were divorced were less likely to start the ART compared to those who were single or never married (HR=0.5; 95%CI=0.3–1.0; p=0.04).
		(*b*) Report category boundaries when continuous variables were categorized	N/A	-
		(*c*) If relevant, consider translating estimates of relative risk into absolute risk for a meaningful time period	N/A	-
Other analyses	17	Report other analyses done—eg analyses of subgroups and interactions, and sensitivity analyses	4	HIV stages at recruitment was the only significant predictor found for failure

				to remain in the treatment.<br><br>In total 81.82% of those who tested for VL achieved viral suppression (intervention: 9 out of 12; control: 18 out of 21). The intervention group was slightly less likely, although not significant, to be virally suppressed in comparison to the control group.
		Discussion		
Key results	18	Summarise key results with reference to study objectives	4	Our analysis further showed that receiving MI counselling significantly increased ART initiation among PWID.
Limitations	19	Discuss limitations of the study, taking into account sources of potential bias or imprecision. Discuss both direction and magnitude of any potential bias	5	Considering the limited number of PWID in Jakarta and Bandung, we needed to adjust the eligibility criteria by including the previously treated patients in addition to those who were ART naïve. Limited number of PWID also made it not possible for us to do randomization.
Interpretation	20	Give a cautious overall interpretation of results considering objectives, limitations, multiplicity of analyses, results from similar studies, and other relevant evidence	5	These results might imply that MI counselling did not sufficiently help the participants to remain in the treatment nor to achieve viral suppression. This finding is related to the nature of PWID that most of them still have issues with their addiction that might be a barrier to stay on treatment.<br><br>As such, these findings also suggest that the benefits of MI counselling may be enhanced by increasing the frequency of the sessions provided. Indeed, it has been suggested previously that a higher number of sessions is associated with better outcomes in behavioural changes, including ART adherence. Nevertheless, it is necessary to evaluate this further in future research.
Generalisability	21	Discuss the generalisability (external validity) of the study results	5	The positive effect of the counselling on ART initiation provides an insight

				about the possibility of its implementation.
Other information				
Funding	22	Give the source of funding and the role of the funders for the present study and, if applicable, for the original study on which the present article is based	5 & 2	

*Give information separately for cases and controls in case-control studies and, if applicable, for exposed and unexposed groups in cohort and cross-sectional studies.

Note: An Explanation and Elaboration article discusses each checklist item and gives methodological background and published examples of transparent reporting. The STROBE checklist is best used in conjunction with this article (freely available on the Web sites of PLoS Medicine at http://www.plosmedicine.org/, Annals of Internal Medicine at http://www.annals.org/, and Epidemiology at http://www.epidem.com/). Information on the STROBE Initiative is available at www.strobe-statement.org.

Supplementary Table 2. Template for Intervention Description and Replication (TIDIeR) MI Intervention.

Brief Name (1)	A Counselling with Motivational Interviewing Approach to Increase Treatment Uptake & Adherence (the MI Counselling)
Why (2)	Motivational Interviewing (MI) is a client-centred approach that strengthens clients' motivation and commitment to change their behaviour. The counsellors will guide the clients toward change, but the clients are the centre of the intervention, the clients are encouraged and empowered to share their barriers of doing something and therefore to find solutions and make a decision for their situations. Since it is simple and brief, the MI counselling can be integrated in healthcare facilities.<br>In Jakarta & Bandung, the MI counselling was implemented as an intervention to support People Who Inject Drugs (PWID) in complying with HIV treatment.
What (Materials) (3)	The MI intervention was delivered following a module that has been developed for this research study. The module was divided into two parts.<br>Part 1: for clients in early stages of change (pre-contemplation, contemplation, and preparation).<br>This part described the five MI principles:<br>Express empathy through reflective listening.<br>Develop discrepancy between clients' goals or values and their current behavior.<br>Avoid argument and direct confrontation.<br>Adjust to client resistance rather than opposing it directly.<br>Support self-efficacy and optimism.<br>Topics were delivered in several sections, as follows:<br>Section 1: Understanding the stages of change: pre-contemplation, contemplation, preparation.<br>Section 2: Medication adherence and risk behaviours.<br>Section 3: Psychological impacts of non-adherence to antiretroviral treatment.<br>Section 4: Psychological impacts of substance use and methadone.<br>Section 5: Improving the treatment adherence and developing alternative behaviours to reach this goal.<br>Section 6: Expressing concern about the treatment adherence.<br>Section 7: Understanding personal values.<br>Section 8: Ambivalence about behaviour changes.<br>Section 9: Relationship with others and the environment.

Section 10: Social responsibility.

Section 11: Self-confidence in & high-risk situations for the treatment adherence.

Section 12: Problem solving.

Section 13: Goal setting & planning.

Section 14: Review and termination.

Part 2: for in later stages of change (action and maintenance) with necessary skills to act or to perform positive behaviours to improve medication adherence and maintain their actions.

Topics were delivered in several sections, as follow:

Section 1: Understanding the stages of change (Action, Maintenance).

Section 2: Identifying issues related to medication adherence.

Section 3: Stress management.

Section 4: Acknowledging achievements.

Section 5: Effective communication.

Section 6: Effective refusal skills.

Section 7: Dealing with criticism.

Section 8: Thought management.

Section 9: Dealing with temptations to stop taking the medications.

Section 10: Ways to enjoy life.

Section 11: Future planning.

Section 12: Self-liberation.

Section 13: Social support.

Section 14: Need assessment and resource identification.

Section 15: Review and termination.

A simplified version of the module was created in a calendar format. The calendar guided the health workers when delivering the MI. It was placed on their desk and, thereby, they could refer to it anytime when they performed the MI counselling.

The materials used:

The calendar (the simplified MI module).

Pen or pencil.

The MI informed consent form.

The MI forms[a]:

The Depression, Anxiety, Stress Scale-21 (DASS-21)

The HIV Adherence Self-Efficacy Scale (HIV-ASES)

The Social Support Network Questionnaire (SSNQ)

The HIV knowledge questionnaire

The Self-report adherence form

The counselling notes

The pill count form

The monitoring tools, which were developed for the purpose of the current study to monitor and evaluate the procedure of the MI intervention. One of the process being evaluated was the MI fidelity:

Was the MI given by the trained (appointed) health workers?

Did the health workers follow the MI stages?

	Have there been any changes in staffing (the health workers)?
	Have any protocol compliance issues been detected?
	The tool was used during the monthly meeting aimed at assessing the MI intervention process.
What Procedures (4)	Study procedures included targeted training to health workers and intervention delivery to clients.
	Health worker training
	The health workers who would deliver the MI counselling were required to participate in an MI training, followed by in-house trainings. Health worker training was provided via an in-person group training session for all health workers participating in the study. This was followed by weekly in-house training for 1 month, with ongoing technical support as needed for the entire study duration.
	Providers of trainings: the research team.
	Recipients of trainings: three doctors, one peer supporter, three HIV counsellors, and a psychologist.
	Two-day intensive group training
	The MI training was held on 29 and 30 August 2017 at Universitas Padjajaran, Bandung. The process of the training was video recorded by a professional team.
	Before starting the first topic of the training, there was a session describing the current research study and the formative research phase performed previously (Januraga, 2018).
	A module was developed explaining a step-by-step process and topics of the training:
	Basic Counselling Skills
	The MI Concept and the Stages of Change
	Assessment Forms of each MI session
	The MI module (first part) and associated case studies
	The MI module (second part) and associated case studies
	These topics were delivered over two days; the first day covered topic 1 to 4 and the second day discussed topic 5. The training participants' knowledge was assessed using pre- and post-tests. In such tests, they answered 20 yes-or-no-questions in 15 minutes. In addition, a technical preparation for commencing the MI intervention was discussed at the end of the second day.
	Weekly in-house training
	After the two-day intensive group training, a weekly in-house training was performed in each intervention site from 9 October to 9 November 2017. In such training, the health workers practiced delivering the MI counselling under a supervision of a psychologist. One psychologist was appointed for each city. The psychologist for Jakarta involved in the development of the modules and was one of the trainers in the MI training, while the psychologist for Bandung followed the MI training as the participant. The MI counselling was performed as a role-play or with a patient who needed it but did not participate in the current study. The psychologist provided feedbacks for the health workers to improve their competence in delivering the MI counselling. Throughout the duration of the study, the psychologists continued to provide technical assistances for the health workers and to supervise them.
	MI Intervention delivery
	The MI counselling was delivered following the MI module. The sections were given depending on the clients' need and ART status. Assessments were performed before initiating the counselling to determine the section that needed to be delivered.
	Baseline Measurement
	Before starting the first counselling session, the clients were asked to fill in several forms as baseline measurements. The questionnaires were slightly different for each client depending on their ART status:
	All clients: the DASS-21 (Depression Anxiety Stress Scale), the SSNQ (Social Support Network Questionnaire), and the HIV knowledge questionnaire.
	The clients who previously lost to follow up after ART initiation and currently re-engaged with care: the HIV ASES
	The client who were already established on ART: the HIV ASES and the self-report ARV adherence questionnaire.
	After filled in all required forms, a date for the first session was agreed between the client and the health worker. Alternatively, they were allowed to start the MI session directly after finishing the baseline measurement.

For all participants, the first MI session started with the section 1 of the MI module Part 1. Other topics would depend on the results of the baseline measurement and the participants' ART status. At the beginning of every session following the first one, the client's psychological condition (depression, anxiety, stress), social support, and HIV knowledge were re-assessed to determine the section that needed to be provided.

Pre ARV treatment initiation

The MI counselling started with Part 1 of the module: Section 1 (Understanding the Stages of Change: pre-contemplation, contemplation, preparation), Section 2 (Medication Adherence and Risk Behaviours), Section 6 (Expression Concern about ART Adherence), and Section 10 (Social Responsibility). These sections were repeated as needed until the clients initiated the ART. There was no requirement concerning the number of the MI sessions to deliver such sections. However, if after repeating them for several times the client did not start the ART, the health worker discussed with the psychologist and the research team to consider the appropriateness of the MI counselling for the particular client.

The clients under this category included:

The clients who were ARV treatment naïve:

The newly diagnosed clients: during the post HIV test counselling they were offered to participate in the current study to receive the MI counselling.

The clients who were previously diagnosed but have never initiated ART.

The clients who previously lost to follow up after ART initiation and currently re-engaged with care.

For these clients, the MI counselling aimed at providing information about the importance of ART. Those who were newly diagnosed also were helped to accept the HIV test result.

Using the MI counselling, those who previously lost to follow up after ART initiation were encouraged to re-initiate the treatment. Their self-efficacy in complying with ART were assessed (using the HIV ASES questionnaire). Those who had a low score, meaning low self-efficacy, were given the topics from Part 1 of the MI module, namely Expression concern about ART adherence (section 6), Understanding personal values (section 7), Ambivalence about behaviour changes (section 8).

ART Initiation

The clients under this category were those who were currently on ART, including those who were already established on ART when they were recruited and recent initiators (who initiated ART during the MI intervention). For such clients, the MI counselling focused on emphasizing the information related to the ART adherence and assisting them to create a plan concerning the adherence.

Those who were already established on ART when they were recruited filled in the DASS-21, the SSNQ, and the HIV knowledge questionnaire. The MI counselling were given following the same rules as mentioned above. However, the sections were repeated until the participants reach better results of each measurement.

The treatment adherence of all clients who were on ART was assessed using a self-report questionnaire. If they did not take ART for two days or more, then the counselling was delivered following the section 3 (Psychological impacts of non-adherence to antiretroviral treatment), section 4 (Psychological impacts of substance use and methadone), section 5 (Improving the treatment adherence and developing alternative behaviours to reach this goal), and section 12 (Problem Solving) of the MI module Part 1 and the section 1 (Understanding the stages of change: Action, Maintenance) of the MI module Part 2. In addition, their self-efficacy in complying with ART was also assessed using the HIV ASES questionnaire. Those who had a low score received the MI counselling based on the MI module Part one: section 6 (Expression concern about ART adherence), section 7 (Understanding personal values), section 8A (Ambivalence about behaviour changes), section 11 (Self-confidence in & high-risk situations for the treatment adherence), and section 13 (Goal Setting and Planning).

ART Maintenance

This category was for the clients who were already established on ART and in the level of change of maintenance. It means that they scored high on the HIV ASES and the self-report adherence (0 to 1 missed dose day). Therefore, in the MI counselling both parts of the module were given, in particular section 14 of Part 1 (Review & Termination), section 2 (Identifying issues related to medication adherence) and section 11 (Future Planning) of Part 2.

Who Provided (5)

The health workers provided the MI counselling. They were medical doctors, nurses, and counsellors, who worked at the HIV/AIDS or harm reduction clinic of the intervention sites. They were appointed by the head of each site to participate in the current study. Most of them received other counselling skill trainings (e.g. basic and HIV counselling skills) before participating in the current study and have been doing counselling as part of their clinical work.

All health workers were required to follow the Two-day intensive group training and in-house trainings prior to the initiation of the MI intervention. In delivering the MI counselling, the health workers were supervised and assessed by the appointed psychologists. Monthly monitoring & evaluation and case conference were performed in each intervention city throughout the

	duration of the study. The case conference provided an opportunity for the health workers to discuss a difficult case. The case conferences were facilitated by the research team and the psychologists.
How (6)	The MI session was an individual face-to-face meeting between the health worker and one client.
Where (7)	The MI sessions were delivered at the intervention sites of the current study, which included:<br>Jakarta: two primary health care facilities (Puskesmas Senen and Puskesmas Grogol Petamburan).<br>Bandung: an outpatient HIV clinic in a general hospital (Teratai clinic).
When & How Much (8)	The MI intervention was performed from 9 January 2018 to December 2019. The clients were required to follow a minimum four and a maximum 10 of the MI sessions within 12 months. Depending on each client's needs, the duration of one session varied between 15 and 60 minutes, and every session consisted of two or more MI sections from either Part 1 or Part 2 of the MI module. There was no requirement of how frequent the sessions should be performed. The health worker and the client were allowed to decide their preferred schedule.
Tailoring (9)	The results of the baseline measurement determined the client's stage of change and the appropriate MI section that would be delivered in the first session. The client can be at any stage of change to start the MI counselling. At subsequent sessions, they were re-assessed before deciding which module section to be delivered.<br>Telephone sessions might be conducted for the clients who were unable to go to the clinic, except for the first session. There were three telephone sessions in Jakarta.
Modification (10)	Staffing:<br>The health workers who delivered the MI counselling:<br>Jakarta:<br>Puskesmas Senen: one doctor was replaced by a nurse because she had other responsibilities given by the head of the Puskesmas.<br>Puskesmas Grogol Petamburan: an additional health worker (a nurse) joined after the two-day intensive group training was held.<br>Bandung:<br>one doctor was replaced by an HIV counsellor because she was appointed as a coordinator of other interventions in the hospital.<br>Training:<br>The additional health workers received a one-on-one training provided by the psychologists. All but one also joined the in-house trainings.<br>Another training was organised in the middle of the intervention period to support the health workers in improving their MI competences. The training was delivered by a psychologist, who was a certified MI trainer. The additional health workers also participated in this training.
How well (planned) (11)	The fidelity of the counsellors in delivering the MI intervention was assessed by the psychologists. The psychologist supervised several MI sessions performed by the health workers and documented any issue, including fidelity.<br>In addition, monthly meeting was performed by the research team to evaluate the process of the MI intervention. The assessment was performed using the monitoring tool.
How well (actual) (12)	In general, the health workers followed the MI principles. Although it took times, it was obvious that the health workers have been becoming more client-centred in providing the MI counselling and interacting with the clients.<br>The major problem documented was the fact that the health workers often forgot the scheduled appointment. The psychologists were responsible to remind them regularly. Another problem was that the health workers, sometimes, were not able to determine the MI section based on the assessments' results. During the first year of the study, the psychologists helped the health workers determine the appropriate section to be delivered and supervised the health workers intensively in providing the MI counselling. After the first year, it was noted that the health workers did not strictly follow the MI module, but they adhered to and applied the MI principles.

[a]The MI forms

- The Depression, Anxiety, Stress Scale-21 measures the client's level of depression, anxiety, and stress, a higher score reflects a more severe condition (normal, mild, moderate, severe)

- The HIV Adherence Self-Efficacy Scale measures the client's level of efficacy in complying with ART, a higher score infers that the client has the ability to comply with ART
- The Social Support Network Questionnaire assess the social support network available for the client
- The HIV knowledge questionnaire evaluates the client's knowledge about HIV and ART, a higher score means that the client has more knowledge about HIV and ART
- The Self-report Adherence form measures the client's level of adherence to ART, a higher score reflects a higher adherence level
- The pill count form is used to document the number of left-over pills during each visit, this data is also used to determine the client's level of adherence (the more the left-over pills, the less adhere is the client to ART)
- The counselling notes were used to document the counselling process, including the client's current stage of changes

Supplementary Table 3. Factors Associated with Failure to Remain in Treatment.

	Failure to Remain in Treatment		Univariate Analysis				Multivariate Analysis			
	n	%	HR	95% CI		p	HR	95% CI		p
**MI Counselling**										
Yes	12	40.0	1				1			
No	31	36.5	1.1	0.6	2.2	0.7	0.9	0.4	1.9	0.8
**HIV Stage at Recruitment**										
Stage 1 and 2	12	25.0	1				1			
Stage 3 and 4	31	46.3	2.1	1.1	4.2	0.03*	2.4	1.2	4.8	0.01*
**ARV Status at Recruitment**										
Naïve	25	32.9	1				1			
Previously treated	18	46.2	1.6	0.9	2.9	0.1	1.8	0.9	3.7	0.1
**Gender**										
Male	41	37.6	1				1			
Female	2	33.3	0.8	0.2	3.5	0.8	1.0	0.2	4.6	1.0
**Age Group**										
Below 35	24	36.9	1				1			
Above 35	19	38.0	1.1	0.6	2.0	0.8	1.2	0.7	2.5	0.5
**Education Group**										
Lower education (junior high school and above)	11	37.9	1				1			
Higher education (high school and above)	32	37.2	1.0	0.5	1.9	0.9	1.1	0.5	2.3	0.9

**Employment**										
Unemployed	16	43.2	1				1			
Employed	27	34.6	0.8	0.4	1.5	0.4	0.8	0.4	1.6	0.5
**Marital Status**										
Single (never married)	16	42.1	1				1			
Currently married	13	27.1	0.6	0.3	1.2	0.2	0.6	0.3	1.3	0.2
Widowed/divorced	14	48.3	1.1	0.6	2.3	0.7	1.0	0.5	2.2	1.0

*significant predictor (p value<0.05)

Supplementary Table 4. Factors Influence Viral Suppression.

	Virally Suppressed		Univariate Analysis				Multivariate Analysis			
	n	%	OR	CI (95%)		p	OR	CI (95%)		p
MI counselling										
No	18	66.67	1				1			
Yes	9	33.33	0.5	0.08	2.99	0.45	1.01	0.12	8.54	0.99
HIV Stage at recruitment										
Stage 1 - 2	16	59.26	1				1			
Stage 3 - 4	11	40.74	0.34	0.05	2.22	0.26	0.16	0.01	1.87	0.14
ARV status at recruitment										
Naïve	23	85.19	1				1			
Was started but then stopped	4	14.81	0.17	0.03	1.19	0.07	0.09	0.01	1.14	0.06
